# Patient perspectives on acromegaly disease burden: insights from a community meeting

**DOI:** 10.3389/fendo.2025.1516131

**Published:** 2025-02-03

**Authors:** Shruti N. Shah, Kevin C. J. Yuen, Vivien Bonert, Wenyu Huang, Jill Sisco, Chrystal Palaty, Kristen Dancel-Manning, Nidhi Agrawal

**Affiliations:** ^1^ New York University Grossman School of Medicine, New York, NY, United States; ^2^ Barrow Neurological Institute, Phoenix, AZ, United States; ^3^ Cedars-Sinai Medical Center, Los Angeles, CA, United States; ^4^ Northwestern University Feinberg School of Medicine, Chicago, IL, United States; ^5^ Acromegaly Community, Grove, OK, United States; ^6^ Metaphase Health Research Consulting Inc., Vancouver, CA, United States; ^7^ New York University Langone Health, New York, NY, United States

**Keywords:** acromegaly, pituitary, patient perspective, quality of life, mental health

## Abstract

**Objective:**

A profound mismatch between biological and symptom control in acromegaly creates a high disease burden despite achieving optimal biological control. There is a great need to learn more about the perspectives of patients living with acromegaly.

**Methods:**

Acromegaly Community hosted a virtual meeting in January 2021 and prepared a detailed report capturing participants’ input on acromegaly symptoms and current and future treatment approaches. The findings of this report are reviewed and summarized in this study.

**Results:**

Fatigue/muscle weakness (92%) and joint pain/arthritis (90%) are the two most common and troublesome symptoms reported by meeting participants. Acromegaly negatively impacts all aspects of daily living: social interaction (49%); exercise (42%); sports/recreational activities (39%); household activities (38%); attending school or job (38%); family relationships (33%); and walking (26%). Anxiety/depression is experienced by 75% of respondents. Eighty-three percent of patients underwent pituitary surgery, and over 71% of patients require medical therapy. Patients desire future improvements in medication efficacy, tolerability, and administration; mental health resources for themselves and their families; and other multimodal approaches to address their physical symptoms, specifically hunger, weight gain, muscle weakness, and joint pains.

**Conclusion:**

Acromegaly patients experience significant physical and psychological burdens despite biochemical control, highlighting the need for comprehensive and patient-centered care. In particular, the impacts on activities of daily living (ADLs) and heavy psychosocial and socioeconomic burdens are striking. We advocate for periodic screening for impacted ADLs, multidisciplinary teams to proactively address these symptoms, and call for further research on under-evaluated aspects of the disease.

## 1 Introduction

Acromegaly is a rare and insidious disease resulting from excessive growth hormone (GH) production, most frequently from a GH-secreting pituitary adenoma. The increased levels of GH and consequently insulin-like growth factor I (IGF-I) have wide-ranging systemic manifestations and clinical comorbidities (1–3). Physical disfigurement and other cardiovascular, respiratory, musculoskeletal, and metabolic complications are well described, but the psychological burden of this debilitating disease is under-recognized and frequently overlooked. Furthermore, physical disfigurement in acromegaly often causes impaired quality of life (QoL), self-perception, and body image (4, 5). Patients with acromegaly also frequently report mood changes resulting in depression, anxiety, and anger issues (6) The discrepancy between symptoms and biochemical control leads to persistent disabilities years after biological remission has been achieved (7–9).

Patient perspectives on pituitary disease and treatment burden are often at odds with provider perspectives (9, 10). Of the pituitary adenomas, acromegaly has been shown to demonstrate the most impaired QoL despite biochemical remission (11–13). Thus, investigating patient perspectives on the lived experience with acromegaly is extremely crucial for patient wellbeing.

To learn more about patients’ perspectives of living with acromegaly, Acromegaly Community hosted a virtual Externally Led Patient-Focused Drug Development (EL-PFDD) meeting in January 2021 for people with acromegaly and their family members, caregivers, and friends; industry, advocacy, and regulatory representatives; scientists; and healthcare providers (14). The findings from this meeting provide a valuable opportunity to learn more about acromegaly directly from those most affected by the disease and their caregivers, whose perspective are also often neglected. We aim to study patient perspectives from the meeting in depth and identify the symptoms that are most burdensome to patients; activities of daily living most impacted; and future treatment directions for physicians to provide more holistic and personalized care to their patients with acromegaly.

## 2 Methods

### 2.1 Attendee outreach

Acromegaly Community is a non-profit patient advocacy organization and online patient support community with nearly 3000 members from around the world. Members of the organization include patients and their family members. The EL-PFDD meeting was primarily advertised to members of the Acromegaly Community Facebook group. Additionally, other patients, healthcare providers, industry professionals, and researchers were informed about the event through other pituitary disease support groups and news channels such as Pituitary World News.

### 2.2 Structure of patient-focused meeting

This EL-PFDD meeting was modeled after the work of the U.S. Food and Drug Administration (FDA) PFDD initiative (15). The meeting began with a talk defining the FDA’s role in facilitating the development of safe and effective medical products for acromegaly. Attendees were then provided with a clinical overview of the biology of acromegaly.

The morning session covered *Living with Acromegaly: Symptoms and Daily Impacts* and aimed to explore the patient and caregiver experience of living with acromegaly, the symptoms that are most significant to individuals with acromegaly, and the impacts of these symptoms on their daily lives. Pre-recorded panel sessions were presented in which five patients shared their experiences regarding the symptoms of acromegaly, and the impacts on their daily life and their fears and worries of living with the disease for the rest of their lives. Participants were then invited to join the discussion via online polling, calling in by phone, or submitting written comments through the online portal.

The afternoon session addressed *Perspectives on Current and Future Approaches to Treatment* and explored the most important treatments and modalities used to manage acromegaly, treatment trade-offs and effectiveness, and preferences for future treatments. The afternoon session began with a presentation on the current and future treatments for acromegaly. A panel of five patients living with acromegaly and caregivers joining via video conference described the different medical interventions they used to treat their disease and other approaches they used for symptom management. Participants were again encouraged to join the discussion via online polling, calling in by phone, or submitting written comments through the online portal.

The online comment submission form for free-text responses was open for 30 days before and after the meeting.

### 2.3 Study procedures and analysis

A 93-page report was prepared by the Acromegaly Community (16), describing participant input during the meeting and in the associated online submission form. We reviewed the report and analyzed the key findings.

#### 2.3.1 Online polling

All online polling questions were only administered to those attendees that were patients with acromegaly. Descriptive statistics for categorial variables determined by online polling are presented as percentages.

Patients were first asked to identify their location (US Eastern Time, US Central Time, US Mountain Time, US Pacific Time, US Alaska Time, US Hawaii Time, Canada, Europe, Middle East, Mexico, Asia, other); gender (male, female, other); current age (<18 years of age, 18-30 years of age, 31-50 years of age, 51-60 years of age, > 60 years of age); age of onset of acromegaly symptoms (<5 years of age, 6-18 years of age, 19-30 years of age, 31-50 years of age, 51-60 years of age, >60 years of age); and age of diagnosis (<5 years of age, 6-18 years of age, 19-30 years of age, 31-50 years of age, 51-60 years of age, >60 years of age).

Next, patients first selected acromegaly-related health concerns they currently experience (patients were able to select all options that apply), and then the top 3 health concerns they find the most troublesome from the same list of options. The options were: fatigue/muscle weakness, joint problems/arthritis, enlarged hands or feet, anxiety/depression, soft tissue swelling, headaches, excessive sweating/body odor, dizziness/vertigo, sleep apnea, vision problems, reproductive system issues (infertility, sexual dysfunction, and uterine fibroids), respiratory problems, type 2 diabetes, cardiomyopathy/heart problems, and other.

Next, patients were asked to identify which specific ADLs which were most affected secondary to acromegaly. Patients were asked to select up to 3 choices. The options were: social interaction and participation, exercising, participation in sports/recreational activities, household activities (cleaning, cooking, etc.), attending school or having a job, family relationships, and walking.

Next, patients answered the question, “Do you consider yourself biochemically controlled? Is your IGF-1 within the normal acceptable range for your gender & age?” This was followed by the question, “Do you consider yourself symptomatically controlled?” Patients selected one of the following answers for both question: always, most of the time, sometimes, rarely, never, unsure.

Next, patients selected all treatments that they have recently used to treat their acromegaly (patients were able to select all options that apply for both of the following questions). The first question asked about medical treatment options: surgery, drugs to reduce growth hormone secretion, drugs to block the action of growth hormone, drugs to lower prolactin levels, anti-depression or anti-anxiety medications, experimental medications as part of a clinical trial, other medications, radiation therapy, stereotactic radiosurgery, and proton beam therapy. An additional question asked about adjunctive treatment options: healthy diet, increased exercise, other weight maintenance strategies (i.e., intermittent fasting), decreased exercise, dietary supplements, stress management, therapy/counseling, CBD/cannabis, modifications/accommodations at home, and other.

Finally, patients selected up to 3 signs and symptoms of acromegaly that they feel should be the target of a new acromegaly medication. The options were: fatigue/muscle weakness, joint problems/arthritis, enlarged hands or feet, anxiety/depression, soft tissue swelling, headaches, excessive sweating/body odor, dizziness/vertigo, sleep apnea, vision problems, reproductive system issues (infertility, sexual dysfunction, and uterine fibroids), respiratory problems, type 2 diabetes, cardiomyopathy/heart problems, biochemical/IGF-1 control, enlarged facial features, and other.

#### 2.3.2 Qualitative analysis

Comments submitted by meeting participants by calling in during the meeting were transcribed, and together with all free-form text responses in the online submission form, were compiled to be utilized for the qualitative component of the study. The qualitative analysis we performed was based on a thematic analysis approach (17, 18). A deductive approach using the predetermined framework of the 7 ADLs asked about in the online polling was used to analyze and identify patient experiences related to these domains. A combination of deductive and inductive approach was used to analyze the transcripts for 1) psychosocial/socioeconomic burdens of acromegaly, and 2) the research and healthcare resource priorities of acromegaly patients. During the coding process, the transcripts were then analyzed in their entirety by the same study team member (S.S.) to ensure consistency. Selected quotes are presented in the text below.

## 3 Results

### 3.1 Study participants and demographics

The EL-PFDD meeting was attended by 304 attendees. In addition to input from the participants present during the meeting, there were 145 comments from 104 individuals on the online submission form submitted before and after the conference.

**Figure 1:**
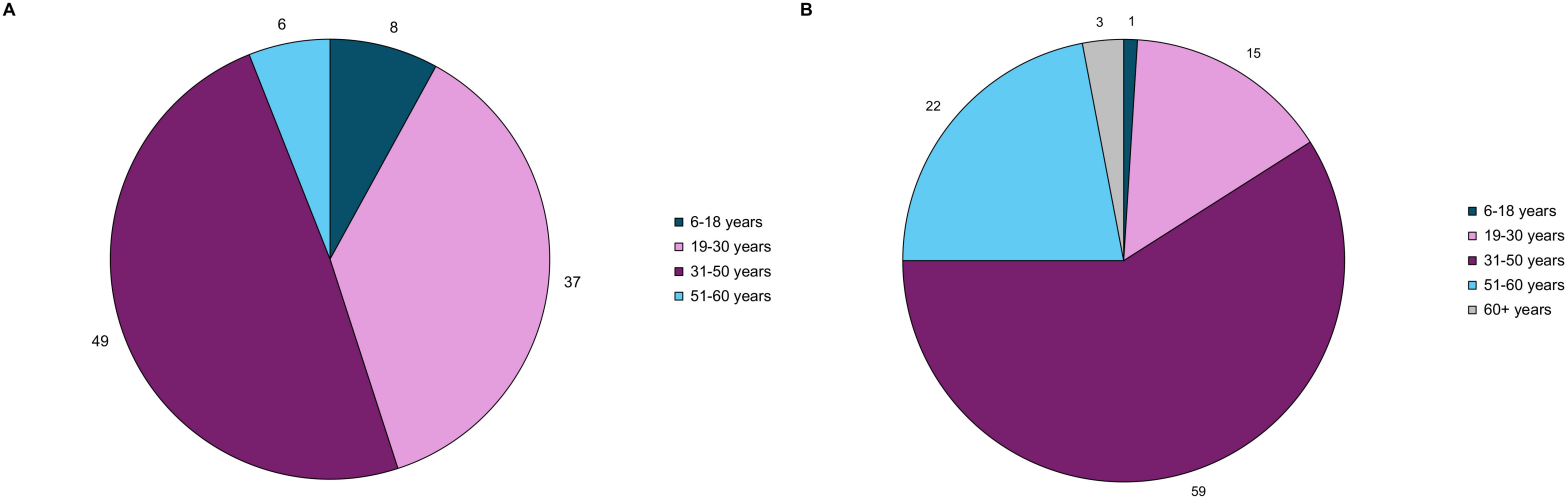
Acromegaly patients (n=79) self-reported age at **(A)** acromegaly symptom onset, and **(B)** acromegaly diagnosis through online polling. These pie charts represent the distribution of age at symptom onset and diagnosis.

Of the 304 attendees, 128 (42%) were patients living with acromegaly. The response rate for the demographics questions ranged from 55-63% of the 128 patients present at the meeting. Online polling revealed the following: 79% (n=55/70) of the respondents were from the US and 74% (n=56/76) were female. One patient (n=76) was below 18 years of age, 7% were between 18-30 years of age, 49% were between 31-50 years of age, 25% were between 51-60 years of age, and 18% were greater than 60 years of age. As seen in **Figure 1**, 8% of patients experienced their first acromegaly symptoms between the ages of 6-18 years, 37% between 19-30 years, about half (49%) between 31-50 years, and 6% between 51-60. The age of acromegaly diagnosis was older, with 1% of respondents diagnosed between the ages of 6-18 years, 15% between 19-30 years, 59% between 31-50 years, and 22% between 51-60 years.

### 3.2 Symptoms and daily impacts of acromegaly

**Figure 2:**
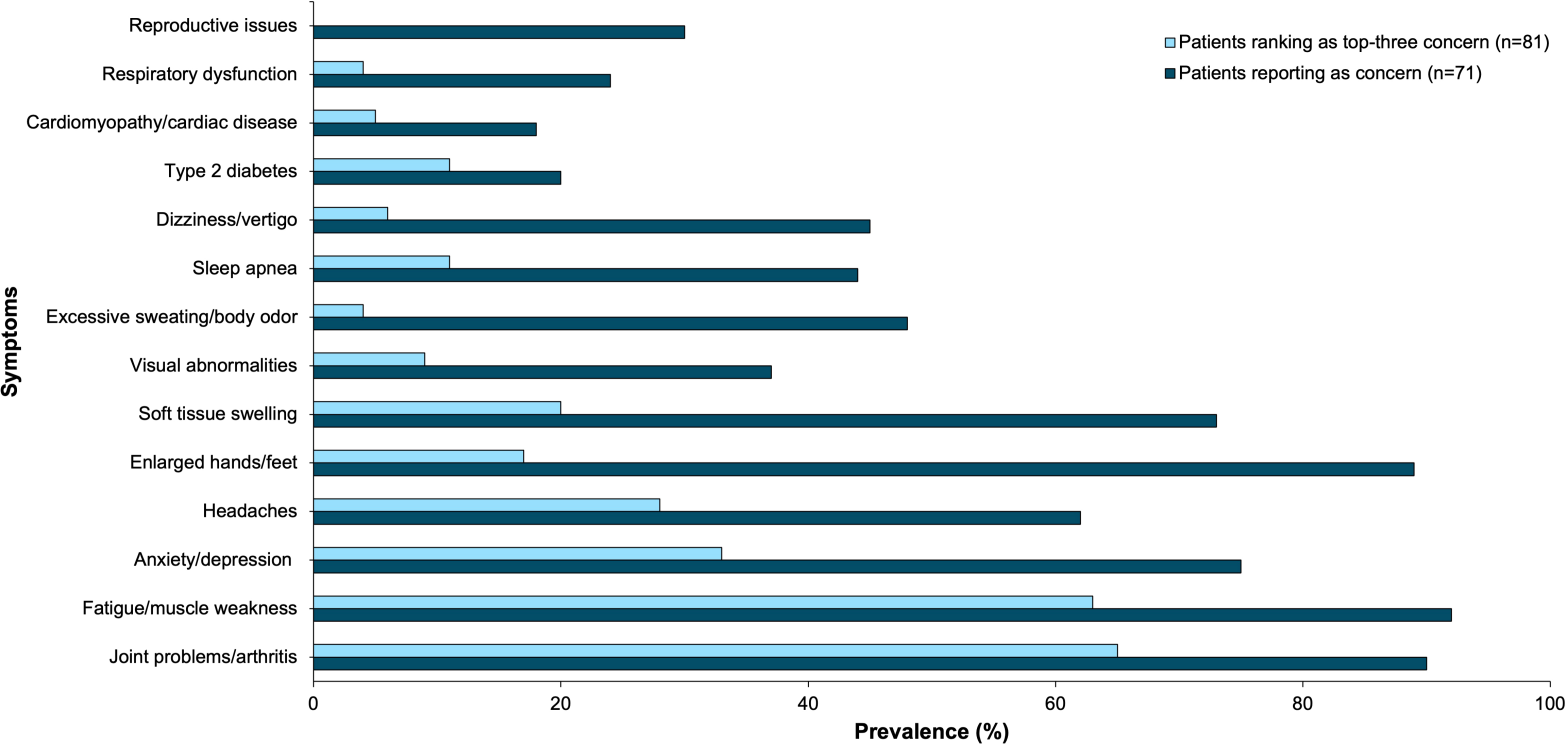
Acromegaly patients self-reported physical and mental health symptoms as concerns (n=71) and top-three concerns (n=81) through online polling. This bar graph represents these symptoms by prevalence.

Fatigue/muscle weakness and joint problems/arthritis were the two most frequently experienced and troublesome health concerns. Ninety-two percent of patients reported experiencing fatigue and muscle weakness, and 63% of patients reported it as a top 3 most troublesome symptom. Ninety percent of patients reported experiencing joint problems/arthritis, and 65% of patients reported it as a top 3 most troublesome symptom. Anxiety/depression was experienced by 75% of participants and was a top-three concern of 33% of participants. The remaining results are shown in **Figure 2**.

**Figure 3:**
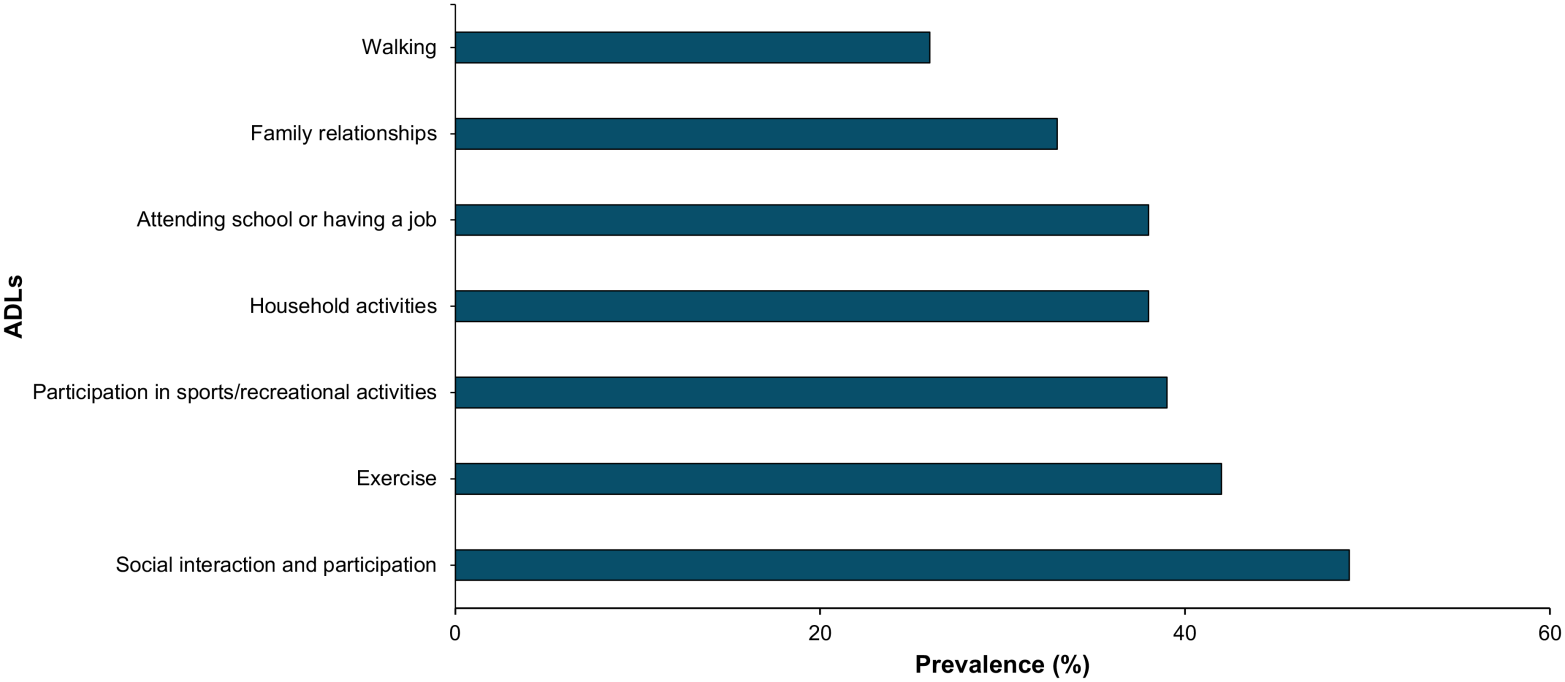
Acromegaly patients self-reported impairment in selected ADLs through online polling (n=69). This bar graph represents ADL impairment by prevalence.

Acromegaly negatively impacts many activities of daily living (ADLs) as seen in **Figure 3**. 49% of respondents reported that acromegaly interferes with social interaction and participation; 42% with exercise; 39% with sports/recreational activities; 38% with household activities like cleaning or cooking; 38% with attending school or having a job; 33% with family relationships; and 26% with walking. Selected patient comments related to these domains are included in **Table 1**.

**Table 1:** Patient experiences related to impaired ADLs.

Impacted Domains	Selected Patient Comments
Social interaction	“Fatigue has also caused me to cut back on socializing, and I know this hurts people that I care about.” “I loved to socialize more but find myself hiding instead because I cannot explain the fatigue and pain that I am in daily.”
Exercise/sports	“Biking/aerobic exercise is out.” “I used to love going dancing, hiking and bouldering, but that is too difficult now.”
Recreation	“I had to stop playing and doing what I was trained most of my life to do, be a clarinetist.” “Gardening and sewing are no longer possible.”
Household activities	“I struggle with simple tasks, such as lifting laundry, carrying groceries, or mowing my lawn and doing simple housework.” “I can’t cook very well for myself because I can’t lift things, I can’t stir things, I can’t chop things.”
Personal care	“I must ask for help buttoning the cuffs for long sleeve shirt or the collared button of a dress shirt.” “I am no longer independent and have become a burden on my family. It is awful.”
Attending school/work	“I had to go on full time disability.” “I ended up losing my job that I adored.”
Family relationships	“I now have irreparable damage to family.” “Because of this condition, I do not feel like I was the wife, mother, or person that I was meant to be.”

**Figure 4:**
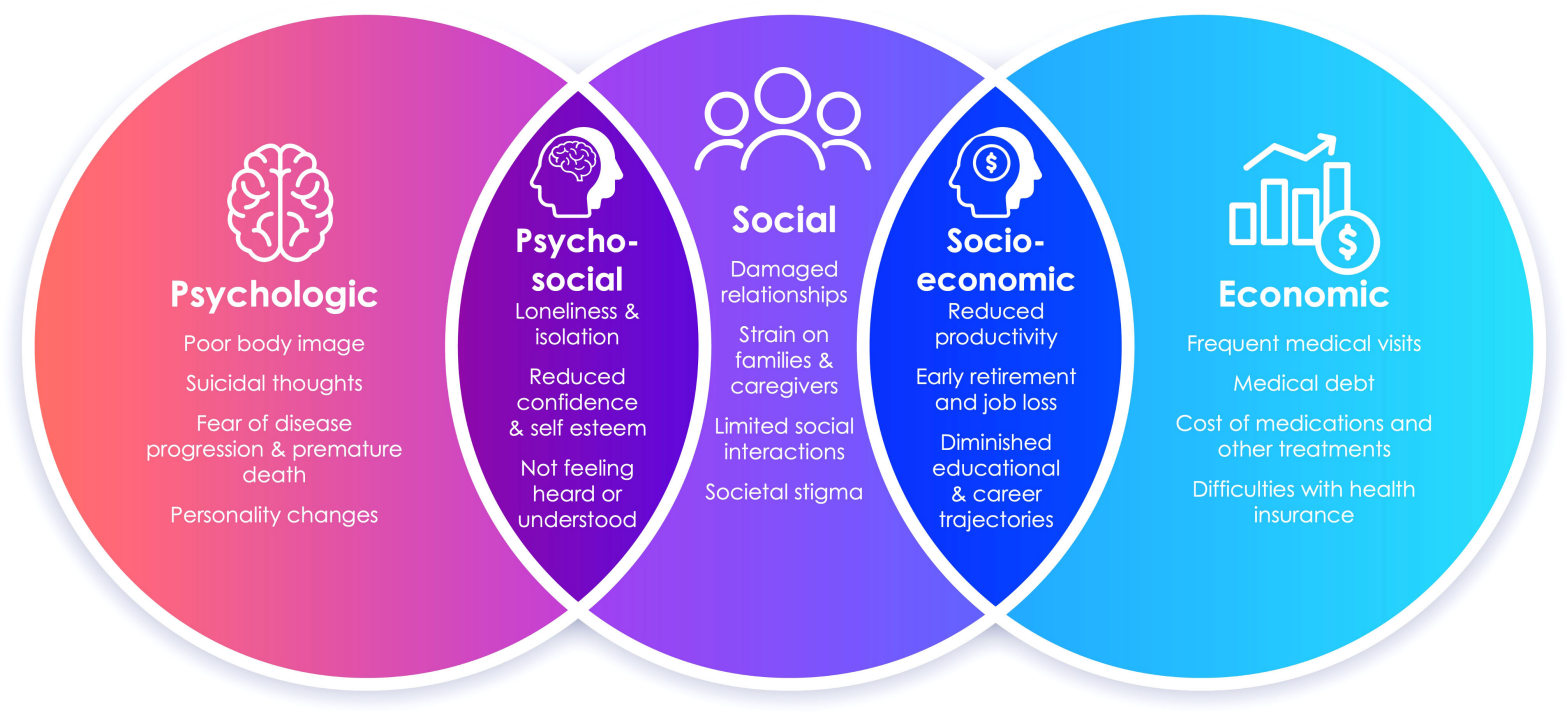
The spectrum of psychosocial and socioeconomic burdens of acromegaly expressed by patients, their family, and other caregivers through free-text responses are displayed as themes and subthemes in this Venn diagram.

Additionally, free-form text responses submitted by patients, their family, and other caregivers allowed us to identify various burdens of acromegaly, displayed in **Figure 4**. Patients described fear of death, damaged relationships, loneliness and isolation, missed career and educational opportunities, and a wide range of financial fears and challenges related to the cost of treatment and the complexity of the healthcare system. As one patient shared, “…contemplating my mortality sets me off into an existential crisis.”

#### 3.2.1 Biochemical vs. symptomatic control

**Figure 5:**
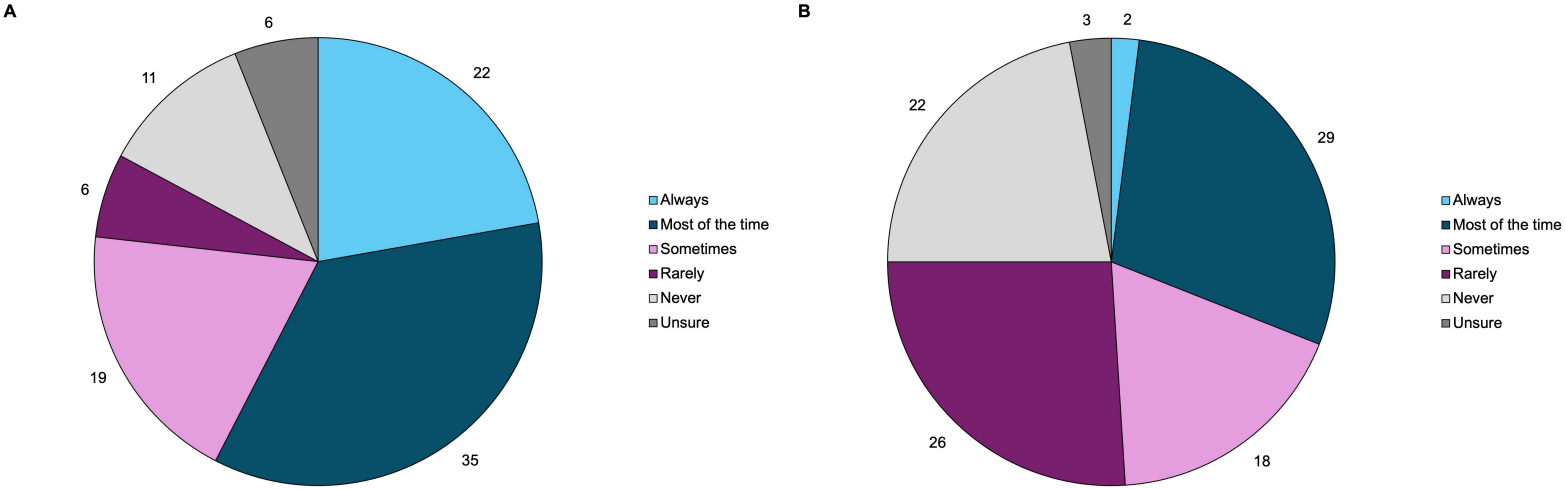
Acromegaly patients self-reported their perception of **(A)** biochemical disease control (n=63), and **(B)** symptomatic disease control (n=65) through online polling. These pie charts represent the distribution of how often patients consider themselves to be biochemically and symptomatically controlled.

Patients self-reported how often they feel biochemically (defined as IGF-1 levels normal for gender and age) and symptomatically controlled, summarized in **Figure 5**. Twenty-two percent of patients feel that they are always biochemically controlled, while only 2% of patients feel that they are always symptomatically controlled. Thirty-five percent of patients feel biochemically controlled most of the time, 19% sometimes, 6% rarely, 11% never, and 6% were unsure. Twenty-nine percent of patients feel symptomatically controlled most of the time, 18% sometimes, 26% rarely, 22% never, and 3% were unsure.

### 3.3 Current treatment approaches

#### 3.3.1 Surgery

**Figure 6:**
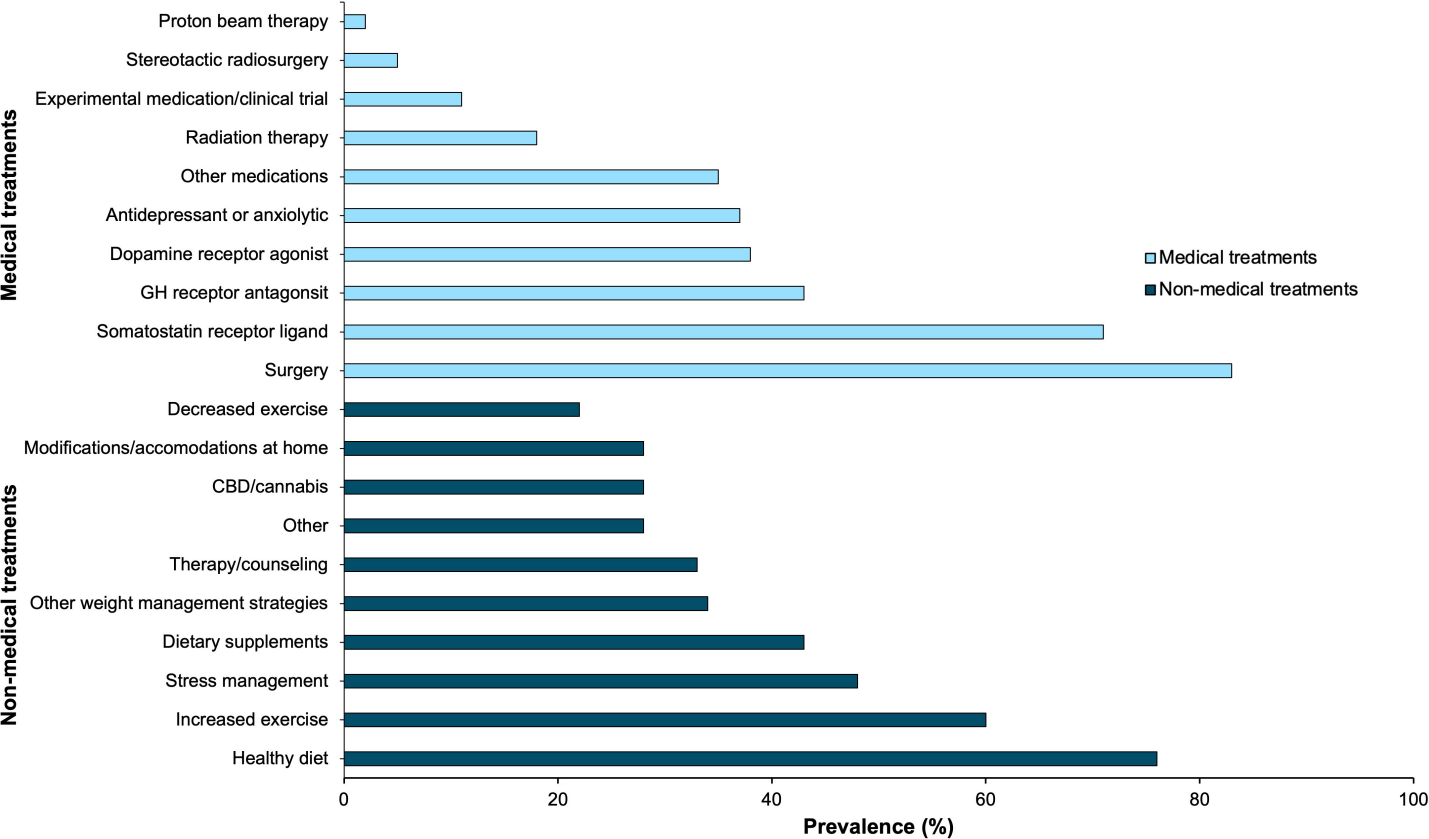
Acromegaly patients self-reported all medical (n=65) and non-medical treatments (n=58) they have recently used through online polling. This bar graph represents these treatment strategies by prevalence.

As seen in **Figure 6**, 83% of acromegaly patients at the meeting underwent surgery (either pituitary or non-pituitary) at some point in time during their treatment course.

#### 3.3.2 Medications

Patients reported use of several different classes of medications; 71% of patients used a somatostatin receptor ligand (SRL), 43% used a GH receptor antagonist, 38% used dopamine receptor agonists, 37% used an antidepressant or anxiolytic, 35% used another medication class not explicitly stated in the polling options, and 11% of patients used an experimental agent as part of a clinical trial at some point during their treatment course (either alone or in combination with other agents).

#### 3.3.3 Radiation therapy

Of respondents, 18% of patients underwent radiotherapy, 5% of patients underwent stereotactic surgery, and 2% of patients underwent proton beam therapy.

#### 3.3.4 Non-medical treatments

As shown in **Figure 6**, 76% of patients implemented a healthier diet, 60% of patients increased their exercise frequency, 48% of patients utilized other stress management techniques, 43% of patients used dietary supplements, 34% of patients implemented other weight management strategies (such as intermittent fasting), and 33% of patients sought therapy and/or counseling. Less common non-medical treatment modalities included CBD/cannabis products, modifications and accommodations at home, decreasing exercise frequency, and other strategies.

### 3.4 Future treatment approaches

**Figure 7:**
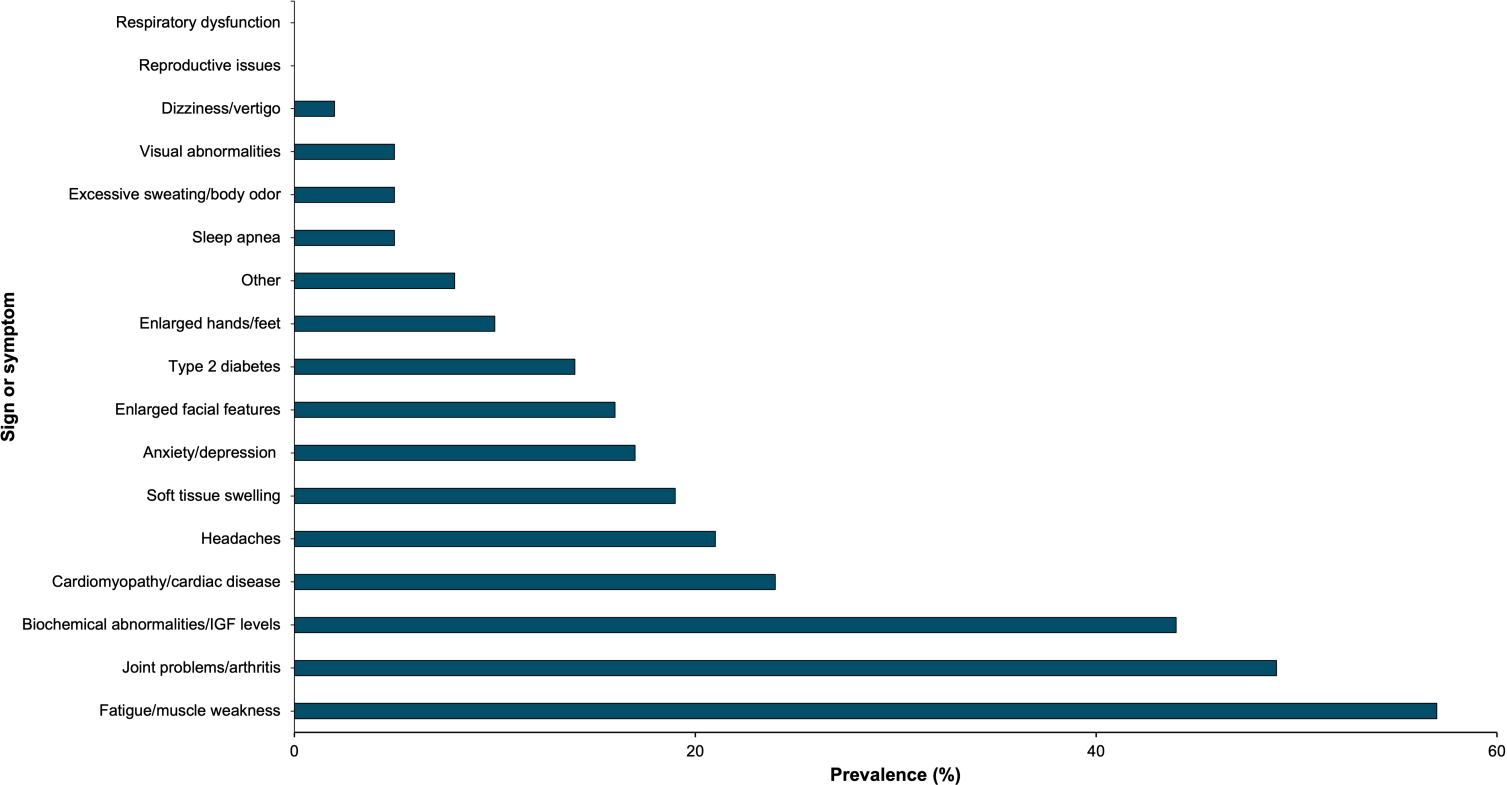
Acromegaly patients (n=63) selected acromegaly signs and symptoms that they consider most important for new drug treatments. This bar graph represents these signs and symptoms by prevalence.

Patients selected up to 3 signs and symptoms of acromegaly that they consider most important if a new acromegaly drug was to be developed. 57% of patients prioritized fatigue/muscle weakness, and 49% of patients selected joint problems/arthritis. Biochemical/IGF-1 control emerged as another popular choice, with 44% of patients selecting this as one of their top three priorities. The remaining results are summarized in **Figure 7**.

### 3.5 Perspectives on future research

Meeting participants shared other treatment and research priorities not captured in online polling. Through thematic analysis of meeting comments, the following sub-themes emerged.

#### 3.5.1 Ease of drug administration

Participants prioritized more effective, tolerable, and easily administered drugs to predictably control their symptoms and IGF-I and GH levels. As one patient noted: “The quality of my life will drastically improve if the daily Somavert injection could be either an oral dose or have a long-lasting effect as to not inject myself every day, it’ll be a game changer. How wonderful it would be to not have the daily painful injections, which leave bruises on my legs and my arms, and are a constant reminder of my incurable disease.”

#### 3.5.2 Monitoring of IGF-1 levels

Additional treatment priorities include lowering IGF-I levels into the reference ranges, greater standardization of IGF-I assays to improve diagnostic accuracy and treatment monitoring, and the development of real-time sensors for GH and IGF-I. One patient wrote, “I wish that normal range would change for Acromegaly patients. The high third of normal range is set too high. Most of us feel best and most pain free when our IGF-1 levels are in the lower third of normal range.”

#### 3.5.3 Hunger and weight gain

Meeting participants expressed their desire to see more research on understanding other biological pathways involved in the pathophysiology of hunger and weight gain in acromegaly, such as ghrelin. One patient wrote, “I believe that we feel hungrier than other people and that this urge to eat must be one of the important factors somehow related to our disease. Most of us struggle to maintain healthy weight. I hope that there will be further research on ghrelin and other hormones which increase and/or control hunger.”

#### 3.5.4 Women’s health issues

Additionally, respondents expressed a desire to see more research on the impact of acromegaly and its medications on salient women’s health issues including menopause, fertility, pregnancy, and breastfeeding. As one patient commented, “I would love to see more research on the effects on these medications on pregnancy and their safety and efficacy during breastfeeding. Making a decision that could impact your unborn child or infant is agonizing.”

#### 3.5.5 Early diagnosis

Respondents at the meeting emphasized the need for earlier diagnosis to reduce acromegaly-related morbidity. One participant commented, “This disease takes away quality of life. I wonder if I was diagnosed earlier, if my old doc really listened and put symptoms together, would I have to deal with tissue damage … colon, heart, joints. I was diagnosed at age 62 and was told I had that tumor for at least 20 years!”

### 3.6 Perspectives on future healthcare resources

Meeting participants expressed their desire to see healthcare resources directed towards improving acromegaly QoL. Through thematic analysis of meeting comments, the following sub-themes emerged.

#### 3.6.1 Public education resources

Another priority expressed by meeting participants was education about acromegaly for the public. A comment read, “It’s very challenging to educate people what Acromegaly is. I think it is such a hard disease to have when no one knows what it is, and you feel so alone.”

#### 3.6.2 Medical education resources

Meeting participants wrote about greater inclusion of acromegaly education in medical school, PA school, and nursing school curriculums to prevent delayed diagnosis and misdiagnosis, among other issues; “health care professionals on all levels need to be educated [that] this disease … is more prevalent than once thought. When a patient presents with symptoms, the health care professional must think about the whole picture and not just focus on the pieces.”

#### 3.6.3 Personal education resources

Additionally, respondents advocated for personal education resources, noting that they have had to educate themselves or rely on peer support groups. One comment read, “I wish that patients would be more educated about diet and effect of different food on Acromegaly. I have learned most facts about this on my own and by comparing experiences with other Acromegaly patients in the Acromegaly support group.”

#### 3.6.4 Mental health resources

Finally, meeting attendees requested greater mental health resources and support for patients, their caregivers, and their family members. One patient wrote, “As a mental health professional, and a person with Acromegaly, mental health issues are at the forefront of my mind. What is being done to address the mental health needs, challenges, and problems of the Acromegaly community?”

#### 3.6.5 National patient registries

Finally, meeting participants expressed a need for the development of a more robust national database or patient registry for acromegaly patients. In terms of patient registries, one patient stated, “The scientific community could look at the possibility to create a large patient registry containing information about how the disease is evolving over time. We still have to learn about the disease. Quality of decisions is related to the quality of information before we make those decisions.”

## 4 Discussion

Our study identifies several critical aspects of the lived experience of patients with acromegaly spanning from physical symptoms to effects on ADLs and their personal relationships. Patients feel that the diagnostic and treatment journey is lengthy and incredibly burdensome, causing frustration for both patients and caregivers. The side effects of both surgical and medical treatments can further complicate recovery. Patients called for earlier diagnosis; greater mental health support and resources; and more emphasis in improving education about acromegaly for patients, medical trainees, and the public.

Meeting participants outlined how more robust national registries would have a positive impact on acromegaly research. This is supported in the literature, with disease registries providing insight into disease trends over time and improving management of chronic disease care (19, 20). Nineteen national acromegaly registries, mostly in Europe, include data from over 16,000 patients (21). Despite their value, limitations such as global underrepresentation, inconsistent data entry, and inconsistent coding accuracy – e.g., a 53% diagnostic predictive value in Danish registries – impact overall registry data quality (21, 22). These gaps emphasize the need for continued investment in enhancing registry infrastructure to advance acromegaly research and establish international standards of acromegaly management.

In our polling, joint problems/arthritis and fatigue/muscle weakness emerged as the most prevalent physical symptoms. They also emerged as the foremost priorities as targets for new development of acromegaly medications. This is in line with prior work that has shown that patients with acromegaly have a high prevalence of arthropathy, muscle weakness, and fatigue even after traditional treatments and even in cases of biochemical remission (23–26).

Patients deal with polypharmacy due to both acromegaly and acromegaly associated comorbidities. In our study, patients frequently required multiple trials and combinations of acromegaly therapy. Fleseriu et al. similarly found that 17.5% of patient required at least three lines of therapy and 16% of patients required combination therapy at some point (27). Medication burden in acromegaly is further complicated by the fact that acromegaly increases the risk of comorbidities and concomitant medication use. In another study, Fleseriu et al. found that acromegaly patients had a significantly higher rate of cardiovascular disorders, and a significantly higher incidence of being prescribed >3 concomitant medications (28). At the meeting, participants expressed a desire for more effective, tolerable, and easily administered medical treatments. Simplifying medication regimens and improving medication tolerability improves medication adherence and consequently patient outcomes and QoL in chronic medical conditions like acromegaly (28–30). Future changes to the treatment landscape and therefore adherence may go a long way in improving disease burden experienced by acromegaly patients.

The pervasive impact of acromegaly on mental health is underscored by the high rates of anxiety and depression in our study, with most patients (75%) reporting these conditions and a third of patients (33%) utilizing anti-anxiety or antidepressants. A similar proportion (33%) of patients also utilize behavioral therapy or counseling. Consistently, Geraedts et al. and Pivonello et al. have found that psychopathology has is one of the strongest predictors of QoL in acromegaly (31, 32). This effect can go beyond just affecting acromegaly patients, with their cohabitants being prone to anxiety and depression as well (33). Targeting mental health conditions remains an area of necessity for adequate management of acromegaly patients. We suggest that emphasizing greater access to mental health care (through both pharmacologic and non-pharmacologic interventions) will benefit patients with acromegaly due to the high prevalence of anxiety and depression.

Patients also felt that delayed diagnosis causes greater physical and psychosocial impairment. This has been studied extensively (34–36), with studies finding that diagnostic delay and lack of diagnostic acumen are strong predictors of poor QoL in patients with acromegaly (37, 38). The long path to diagnosis has also been shown to lead to a loss of trust in medical professionals and self-efficacy (39). Strategies that encourage earlier diagnosis like greater educational awareness of acromegaly among medical trainees and other healthcare professionals would also be to the benefit of acromegaly patients.

Particularly, the strength of our analysis is in the deepening of understanding of which ADLs are most impacted in patients. Previously, studies have found that acromegaly interferes with QoL, with impacts on body image, energy, physical and social functioning, cognition, general health perception, sleep, and sexual function (40, 41). The biopsychosocial model has previously been studied in pituitary disease and has shown that patients may continue to experience disability despite their biological parameters being within the reference range (42, 43). The results of our study implement the biopsychosocial model and highlight impacts on mental health and social interactions and go a step further to highlight impacts on economic domains like employment status and career growth. Economic stability is a crucial social determinant of health and has impacts on treatment efficacy (44, 45); patient providers and care teams would be remiss to ignore impacts of acromegaly on a patient’s livelihood and finances. These findings underscore the urgent need for comprehensive screening and management strategies that address the psychological, social, and economic dimensions of acromegaly.

The study’s findings must be viewed in the context of the small cohort size (304 attendees total, of which only 128 were patients and able to answer online polling questions) and moderate response rate to online polling questions (<63%). Additionally, polling results and comments were collected and reported on jointly for patients with biochemically/symptomatically controlled and uncontrolled acromegaly, making it difficult to assess which symptoms and burdens differentially affect patients with or without disease remission. As only some patients in the Acromegaly Community were motivated to attend the meeting, the participants at this meeting may represent more polarized experiences with acromegaly than the average patient. While acromegaly has a mostly balanced gender distribution (46), 74% of patients that attended this meeting were female. As women are more likely to join support groups than men (47), the Acromegaly Community support group, the largest source of meeting attendees, is likely majority female. Furthermore, infertility is common in reproductive-age patients with acromegaly, and the current lack of clinical guidelines around pregnancy limits the ability of providers to provide evidenced-based recommendations to women around conception and pregnancy surveillance (48–50). Female patients with acromegaly also experience more significant psychosocial distress related to self-esteem and body image (4, 51, 52). The women’s health issues (i.e., fertility and menopause) that emerged as research priorities during the meeting, and large burden of psychosocial distress expressed by attendees, were likely a result of the female-predominant patient population in this study.

Furthermore, several health concerns associated with acromegaly were not explicitly captured in polling. These include chronic pain, changes in facial appearance, impaired cognition, weight gain, gastrointestinal disorders, and pituitary damage or dysfunction leading to secondary adrenal insufficiency (53–55). Future work can explore these areas and their associated impact in greater depth.

Finally, it may be helpful to utilize a disease specific patient-reported outcome measure (PROM) to measure symptom severity [Acromegaly Symptom Diary (ASD), Patient-Assessed Acromegaly Symptom Questionnaire (PASQ) or QoL [Acromegaly Quality of Life Questionnaire (AcroQoL)] in future studies; these tools are well-established in acromegaly and have demonstrated high validity and responsiveness to change (56–61). The use of generic PROMs such as those available via the Patient Reported Outcomes Measurement Information System (PROMIS), could offer another alternative (62). As they are designed to encompass a wide range of health conditions and employs computer-adaptive testing, they may be easier to utilize and particularly valuable in situations where disease-specific PROMs may not fully capture broader aspects of patient experience (60, 62–64). These approaches represent a valuable direction for future studies as they could deepen our understanding of the patient experience in living with acromegaly.

Our findings highlight the necessity for a patient-centered approach that prioritizes multimodal symptom management and QoL improvements. The patients in our study repeatedly emphasized a need for focus on their mental health, physical fitness, diet, and pain control. As suggested by Frara et al. and Casanueva et al., pituitary tumor centers of excellence (PTCOEs), already consisting of neurosurgeons, endocrinologists, oncologists, neuroradiologists, and neuropathologists, may benefit from including psychiatrists, psychologists, physical and occupational therapists, and registered dieticians in the patient care team (10, 65, 66). Due to larger patient volume and more available data, PTCOEs also provide a novel approach to monitor treatment efficacy and applications of clinical guidelines in acromegaly care as reported in a recent study by Giustina et al. (67); these initiatives are a promising step towards establishing international standards of care in acromegaly management based on real-world practice trends.

In conclusion, our study highlights the multifaceted impacts of acromegaly, emphasizing the need for early diagnosis, improvements in medication complexity and tolerability, research into underrepresented realms like fertility, and comprehensive mental health support. The findings underscore the importance of a patient-centered approach that integrates multimodal symptom management and the inclusion of diverse healthcare professionals in the acromegaly care team. Future research should address socioeconomic challenges and other health concerns not captured in this study to further enhance patient care.

## Data Availability

The original contributions presented in the study are included in the article/Supplementary Material. Further inquiries can be directed to the corresponding author.
